# Disentangle a Complex MALDI TOF Mass Spectrum of Polyethylene Glycols into Three Separate Spectra via Selective Formation of Protonated Ions and Sodium or Potassium Adducts

**DOI:** 10.1021/jasms.2c00250

**Published:** 2022-11-09

**Authors:** Xianwen Lou, Joost L. J. van Dongen, Joris W. Peeters, Henk M. Janssen

**Affiliations:** † Laboratory of Macromolecular and Organic Chemistry, Eindhoven University of Technology, P.O. Box 513, 5600 MBEindhoven, The Netherlands; ‡ SyMO Chem B.V., P.O. Box 513, 5600 MBEindhoven, The Netherlands

## Abstract

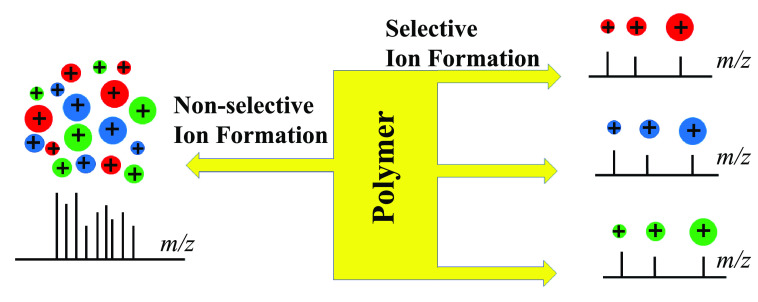

In MALDI TOF MS analysis, complicated mass spectra can usually be recorded for polymers with high affinities to protons and alkali metal ions. For these polymers, protonated ions and sodium and potassium adducts can often be formed concomitantly. By distributing these ions into three separate spectra of protonated ions, sodium adducts, and potassium adducts, significantly simplified spectra can be acquired. Mass spectra consisting of only sodium or potassium adducts can often be obtained by simply adding sodium salt and potassium salt, respectively. We report here a method to selectively generate protonated ions. A polyethylene glycol (PEG) sample with amino end groups was selected as the model polymer and α-cyano-4-hydroxycinnamic acid (CHCA) as the matrix. Octadecylamine (ODA) or a mixture of a tetrabutylammonium (TBA) salt and an ammonium salt was used as the co-matrix to inhibit the release of sodium and potassium ions and their related adducts into the MALDI gas phase plume. By depositing the polymer sample on top of a preloaded layer of CHCA with a co-matrix, the generation of Na^+^ and K^+^ adducts is suppressed, while [ODA + H]^+^ and NH_4_^+^ released from the preloaded matrix layer can serve as protonation reagents to protonate the polymer molecules via proton transfer reactions. It is clearly demonstrated that disentangling a complex mass spectrum filled densely with various series of ions into three separate spectra, with each one consisting of only one type of ions, allows unambiguous identification of mass peaks and greatly helps the interpretation of MS results.

## Introduction

Matrix assisted laser desorption/ionization mass spectrometry (MALDI MS) has not only revolutionized the analysis of proteins and peptides^1,2^ but also provided a powerful and versatile method for the characterization of synthetic polymers.^3−6^ Synthetic polymers are macromolecules consisting of various numbers of repeating units and can be complex mixtures with molecular weight and end-group distributions.^7^ One attractive advantage of MALDI MS for polymer analysis is that ions recorded are normally singly charged, which can greatly simplify the interpretation of the MS results and facilitate the reliable reckoning of monomer and end-group masses.^3−6^ In spite of this, for some polymers with both high proton affinity and high alkali metal ion affinity, such as the polyethylene glycol (PEG) sample studied in this application note, protonated ions and sodium and potassium adducts of the analytes can all be recorded concomitantly. Sodium/potassium salts are even not required to be added intentionally to the MALDI sample, because trace amounts of sodium/potassium impurities ubiquitously present in the sample, glassware, solvents, and reagents are usually sufficient to generate strong signals of the alkali metal ion adducts.^8^ The MALDI MS spectra for these polymers can, therefore, still be very complicated and difficult to interpret depending on the complexity of polymer distributions.

An ideal approach to tackle the complicated mass spectra would be to distribute the ions into three separate mass spectra, with each spectrum containing only protonated ions, sodium adducts, or potassium adducts, respectively. For many oxygen-containing polymers with high alkali metal ion affinity, directly adding a suitable salt of either sodium or potassium in the MALDI sample was found to be a convenient and effective way to form exclusively sodium or potassium adducts of the polymer.^9^ The high alkali metal ion affinity, on the other hand, makes it difficult to generate protonated polymer ions selectively without sodium/potassium adducts. Fortifying the formation of protonated analyte ions by eliminating or reducing sodium and potassium adducts is the subject of many studies on MALDI applications, especially for biomolecules.^10−13^ We report here a method to selectively generate protonated ions for polyglycol samples.

It has been widely recognized that the metal ion adducts are most likely formed in the MALDI gas phase plume.^14^ To eliminate the formation of the alkali metal ion adducts for a polymer, therefore, a practical way is to inhibit the transfer of Na^+^ and K^+^ and their related adduct ions into the gas phase plume. In this application note, a PEG sample was selected as the model polymer. α-Cyano-4-hydroxycinnamic acid (CHCA) was used as the matrix, and octadecylamine (ODA) or a tetrabutylammonium salt was used to suppress the release of Na^+^ and K^+^ and their related adducts.^15,16^ The introduction of co-matrixes has long been used to improve the quality of MALDI measurements.^17,18^ By depositing the polymer sample on top of a preloaded layer of CHCA with a suitable co-matrix using a modified thin-layer method,^19^ selective formation of protonated ions can be obtained for the polymer. The aim of this work is to develop a method that can simplify MALDI MS analysis of complex polymer samples. Evidently, by distributing various ions into three mass spectra with each one containing only protonated ions and sodium or potassium adducts, peak assignment can be greatly simplified and be more reliable, especially in the analysis of a complex polymer sample.

## Experimental Section

### Chemicals

Three polymer samples were used in this study. The samples of polyethylene glycol and polypropylene glycol with amino end groups (PEG–NH_2_ and PPG–NH_2_) were obtained from Sigma-Aldrich (Zwijndrecht, The Netherlands), and the PEG with hydroxy end groups (PEG–OH), from Polymer Laboratories BV (Heerlen, The Netherlands). α-Cyano-4-hydroxycinnamic acid (CHCA) was purchased from Fluka (Zwijndrecht, The Netherlands). Octadecylamine, trihexylamine, ammonium fluoride, diammonium hydrogen citrate, and tetrabutylammonium hexafluorophosphate were obtained from Sigma-Aldrich.

### Sample Preparation

Matrix solutions were freshly prepared in THF or in a mixed solvent of water/acetonitrile (1/1 v/v with 0.1% of trifluoracetic acid) at concentrations of approximately 20 mg/mL. All of the sample solutions were also freshly prepared at concentrations of about 1 mg/mL. Two sample deposition methods were employed in this study, namely, the dried-droplet and modified thin-layer methods.^2,19^ For the dried-droplet method, a sample solution and a matrix solution were mixed in an Eppendorf tube. A 0.5 μL portion of the mixed solution was pipetted onto a stainless steel MALDI plate and allowed to dry. For the modified thin-layer method, a matrix solution and a co-matrix solution were first mixed; then, 0.5 μL of the mixed solution was deposited on the target plate and allowed to dry. After that, a 0.5 μL aliquot of analyte solution in chloroform or water was deposited on top of the first layer and allowed to dry. Chloroform or water was chosen as the solvents for the polymers using the modified thin-layer method with the aim to minimize the embedment of analytes in the matrix crystals.^19^

### Mass Spectrometry

The MALDI TOF MS measurements were performed with an Autoflex Speed (Bruker, Bremen, Germany) instrument. The accelerating voltage was held at 19 kV and the delay time at 130 ns for all experiments. Mass spectra were acquired in the reflector positive ion mode by summing spectra from 500 random laser shots at an acquisition rate of 100 Hz.

## Results and Discussion

**Figure 1:**
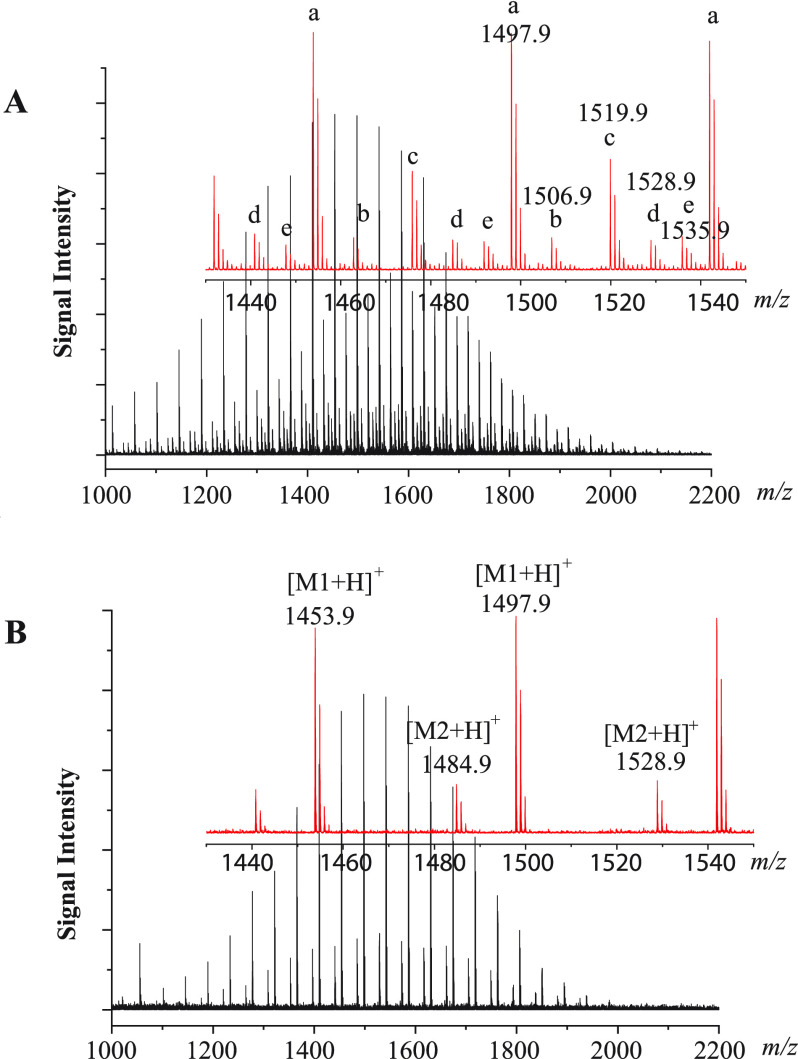
MALDI TOF MS spectra of a PEG–NH_2_ sample. (A) A spectrum obtained with CHCA matrix using the dried-droplet sample deposition method. “a–e” indicate five different series of ions with repeating unit mass of 44 (C_2_H_4_O). The inset in red is a zoomed-in picture of the spectrum. (B) A spectrum with only protonated ions, a sample solution in water deposited on top of a preloaded layer of CHCA with ODA as the co-matrix, CHCA/ODA (5/2 mol ratio). “M1” represents NH_2_C_3_H_6_–(OC_2_H_4_)_*n*_–OC_3_H_6_NH_2_ and “M2” NH_2_C_3_H_6_–(OC_2_H_4_)_*n*_–OH. The inset in red is a zoomed-in picture of the spectrum.

A MALDI TOF mass spectrum of a PEG sample obtained by using CHCA matrix and the conventional dried-droplet sample deposition method is shown in Figure 1A. This PEG sample is supposed to be NH_2_C_3_H_6_–(OC_2_H_4_)_*n*_–OC_3_H_6_NH_2_. PEGs are known to be prone to form alkali metal ion adducts in MALDI. With the amino groups at both ends, it is expected that protonated ions and sodium and potassium adducts will be recorded for this polymer. Major peaks corresponding to protonated ions and sodium and potassium adducts for NH_2_C_3_H_6_–(OC_2_H_4_)_*n*_–OC_3_H_6_NH_2_ can indeed be readily identified. In addition, however, other unknown series of peaks, also with the repeating unit mass of C_2_H_4_O which do not match the given molecular formula, were also recorded. The presence of the unknown impurity peaks in combination with the concurrent formation of protonated ions and sodium and potassium adducts for the polymer makes the resulting mass spectrum rather complicated and difficult to interpret. As Na^+^ and K^+^ are attached to the PEG backbone while H^+^ to the amino end groups, the molecular information that can be deduced for the polymer from the metal ion adducts and protonated ions might be complementary. Therefore, it is practically beneficial to develop MALDI methods that can selectively form protonated ions and metal ion adducts separately for the polymer. In this way, an otherwise complicated MALDI TOF mass spectrum can greatly be simplified by dispensing the polymer ion signals into three separate spectra with solely protonated ions, sodium adducts, and potassium adducts, respectively.

### 1 Selective Formation of Protonated Ions for Polyglycols with Amino End Groups

As ions recorded in a MALDI spectrum are generally singly charged, it was initially anticipated that only protonated ions would be observed if the polymer molecules are completely protonated. In contrast, however, strong signals of Na^+^ and K^+^ adducts could still be observed even when the PEG–NH_2_ sample was dissolved in an acidic solution with 1% trifluoroacetic acid (TFA). Under acidic conditions, both amino groups in the NH_2_C_3_H_6_O–(C_2_H_4_O)_*n*_–C_3_H_6_NH_2_ molecules must be protonated prior to laser desorption. The observation of the Na^+^ and K^+^ adducts must be due to the extremely strong affinity of PEG to the metal ions. In this section, we report two approaches that can selectively form protonated ions for polymers without Na^+^ and K^+^ adducts.

In the first approach, octadecylamine (C_18_H_37_NH_2_, ODA) was used as a co-matrix to prohibit the formation of the alkali metal ion adducts. In a previous article,^16^ we reported that amines can completely suppress the matrix ions including the Na^+^ and K^+^ adducts. Hence, if the PEG sample is deposited on top of a preloaded and dried spot of CHCA mixed with ODA, no Na^+^ or K^+^ adducts for the polymer can be formed because the metal ions and their adducts are not available in the gas phase plume. The details for depositing the PEG sample on top of a preloaded spot of CHCA and ODA are given in the Experimental Section, and the MALDI TOF MS spectrum is shown in Figure 1B. As expected, no Na^+^ and K^+^ adducts are observed for the polymer, and the ions recorded are only the protonated ones which can be assigned as [NH_2_C_3_H_6_O–(C_2_H_4_O)_*n*_–C_3_H_6_NH_2_ + H]^+^ and [NH_2_C_3_H_6_O–(C_2_H_4_O)_*n*_–H + H]^+^. Since the protonated matrix ions which usually serve as the protonation reagents in the gas phase plume were also completely suppressed by ODA, protonation of the PEG molecules was most likely via a proton transfer reaction from [ODA + H]^+^.
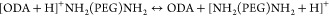
1

Interestingly, when the experiment was repeated with trihexylamine ((C_6_H_13_)_3_N, THA, a tertiary amine) instead of ODA (a primary amine), no protonated ions of PEG could be detected. Similar to ODA, THA can also effectively suppress the formation of matrix ions.^16^ However, to protonate the PEG molecules, protons should be transferred from the protonated tertiary amine of [THA + H]^+^ to the primary amine of NH_2_C_3_H_6_O–(C_2_H_4_O)_*n*_–C_3_H_6_NH_2_, which is thermodynamically unfavorable. The failure of using [THA + H]^+^ to generate protonated ions of the PEG molecules supports the assumption that the protonated PEG ions shown in Figure 1B were formed by proton transfer from [ODA + H]^+^. Based on the discussions above, the functions of ODA are twofold: (1) to inhibit the release of alkali metal ions and their adducts into the gas phase plume and (2) to protonate analyte molecules via proton transfer from [ODA + H]^+^.

It should be noted here that, to effectively suppress the formation of alkali metal ion adducts, the PEG sample should be placed on top of a preloaded and dried spot of CHCA with ODA. Care must be taken to prevent the redissolving of the matrix from the preloaded spot by the PEG solution applied. If the PEG sample is mixed and co-crystallized with CHCA and ODA, ODA can not suppress the metal ion adducts of the polymer and clear Na^+^ and K^+^ adduct signals can again be observed. Most likely, the metal ion adducts of the polymer are formed during the MALDI desorption process and ODA cannot effectively suppress the release of these adducts.

To avoid redissolving of the preloaded layer of matrix, water was used as the solvent for the PEG sample in Figure 1B. However, water is usually not a good solvent for synthetic polymers. Considering the commonly used MALDI matrixes, CHCA is not soluble in chloroform. Therefore, suppressants of Na^+^/K^+^ adducts, preferably insoluble in chloroform, should be potentially useful for MALDI MS analysis of synthetic polymers using the two-layer sample deposition method. Inspired by the successful applications of MALDI MS for the analysis of oligonucleotides,^12^ ammonium salts which are usually not soluble in organic solvents were used to promote the formation of protonated ions and suppress that of alkali metal ion adducts of the PEG molecules. For oligonucleotide analysis, the introduction of an extra amount of ammonium salts can transform the sodium or potassium salts of oligonucleotides into their corresponding ammonium salts, and these ammonium salts will release ammonia and leave a proton on the molecule during MALDI ionization. Among the various ammonium salts tested, ammonium fluoride and diammonium hydrogen citrate (DAC) were found to be the most effective ones for oligonucleotides.^12^ Indeed, by using these two ammonium salts as co-matrixes, the signals of alkali metal ion adducts for the PEG sample decreased significantly but could not be suppressed completely. Further, we also tried the combination of ammonium salts and fucose as co-matrixes, which was found to be more efficient for oligonucleotides,^13^ but still failed to suppress the Na^+^ and K^+^ adducts entirely. The reason for the incomplete suppression is most likely because the ammonium salts and fucose cannot fully inhibit the formation of [CHCA + Na/K]^+^ which will transfer Na^+^/K^+^ ion to PEG. In fact, clear peaks of [CHCA + Na/K]^+^ were observed in the mass spectrum of CHCA with ammonium salts and fucose as co-matrixes.

**Figure 2:**
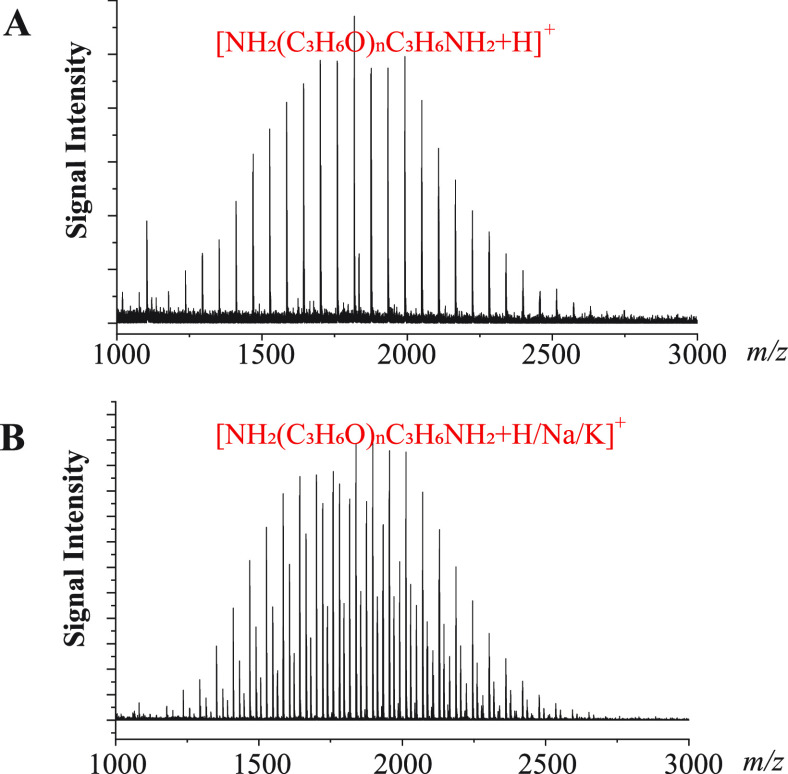
MALDI TOF MS spectra of a PPG–NH_2_ sample. (A) A spectrum with only protonated ions, a sample solution in chloroform deposited on top of a preloaded layer of CHCA with diammonium hydrogen citrate (DAC) and [N(C_4_H_9_)_4_]PF_6_ as co-matrixes, CHCA/DAC/TBAPF_6_ (100/50/1 mol ratio). (B) A spectrum with strong signals of protonated ions and sodium and potassium adducts, using CHCA matrix and the dried-droplet sample deposition method.

In order to generate protonated ions exclusively, [CHCA + Na/K]^+^ must be completely suppressed. It has been reported that tetrabutylammonium (TBA) salts are extremely effective in suppressing all kinds of matrix ions.^15,20^Figure 2A shows a MALDI spectrum of a polypropylene (PPG, NH_2_(C_3_H_6_O)_*n*_C_3_H_6_NH_2_) sample by depositing a PPG solution in chloroform on top of a preloaded layer of CHCA with DAC and tetrabutylammonium PF_6_ (TBAPF_6_). In this way, the matrix ions can be completely suppressed by TBA while NH_4_^+^ can serve as a protonation reagent, leading to the selective generation of protonated ions for the PPG molecules. Similar results were also obtained when DAC was replaced with ammonium fluoride. In contrast, strong signals of protonated ions and sodium and potassium adducts are all recorded when CHCA was used alone (Figure 2B).

### 2 Disentangle a Complex MALDI Spectrum into Three Simplified Ones

**Figure 3:**
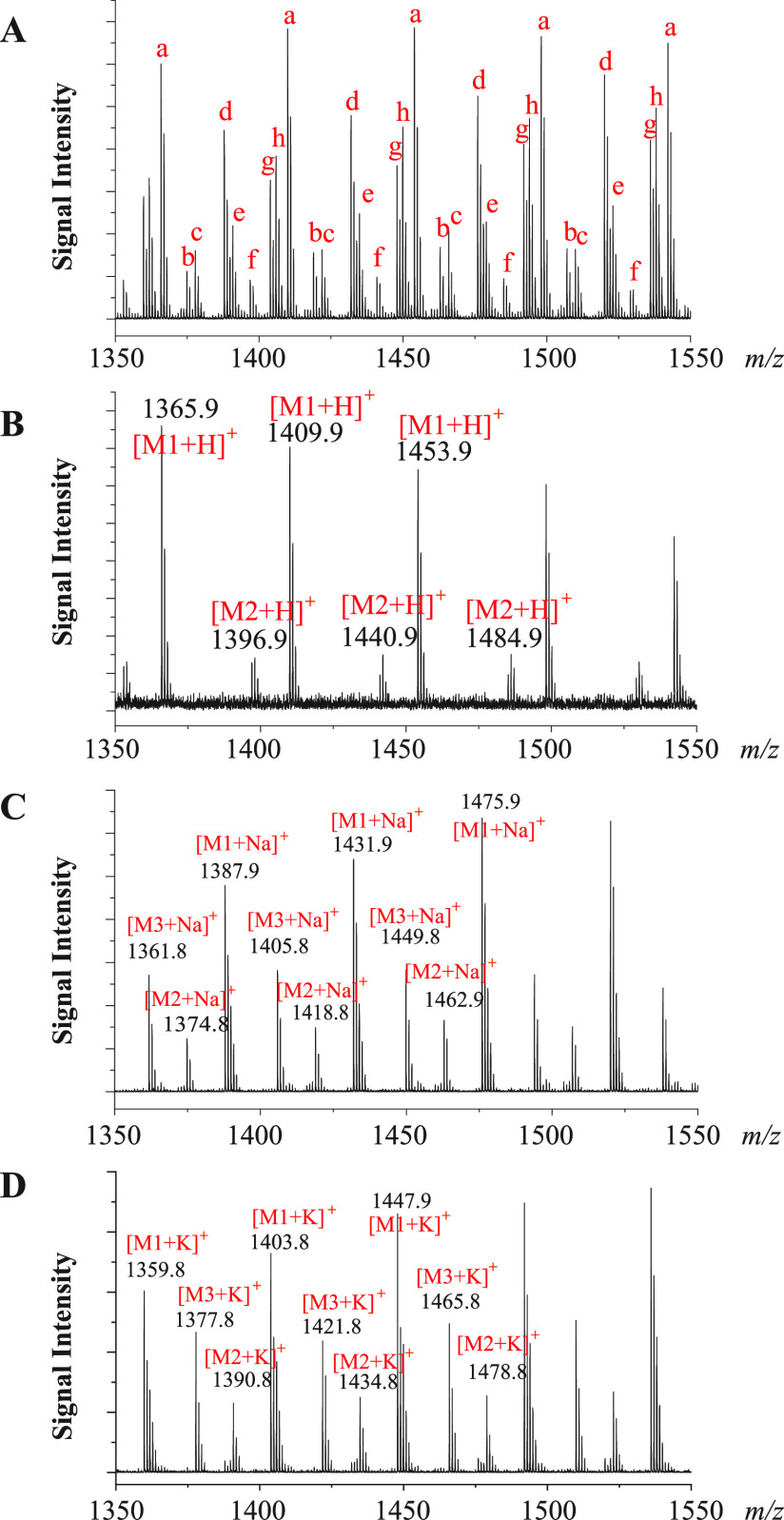
MALDI TOF MS spectra of a mixture of PEG–NH_2_ and PEG–OH. (A) A spectrum of all ion species. The CHCA matrix was deposited using the dried-droplet sample deposition method. “a–h” indicate eight different series of ions with repeating unit mass of 44 (C_2_H_4_O). (B) A spectrum with only protonated ions, a sample solution in chloroform deposited on top of a preloaded layer of CHCA with DAC and TBAPF_6_ as co-matrixes, CHCA/DAC/TBA (100/50/1 mol ratio). (C) A spectrum with only sodium adducts, CHCA matrix with NaTFAc as the co-matrix (CHCA/NaTFAc 200/1 mol ratio). (D) A spectrum with only potassium adducts, CHCA matrix with KTFAc as the co-matrix (CHCA/KTFAc 200/1 mol ratio). “M1” represents NH_2_C_3_H_6_–(OC_2_H_4_)_*n*_–OC_3_H_6_NH_2_, “M2” NH_2_C_3_H_6_–(OC_2_H_4_)_*n*_–OH, and “M3” HO–(C_2_H_4_O)_*n*_–H.

Figure 3A shows a MALDI TOF MS spectrum of a PEG mixture. The spectrum looks very complicated and congested, as it contains numerous species of ions with a repeating unit mass of 44 (CH_2_CH_2_O). These ions can either be protonated ones or sodium or potassium adducts, and they are densely packed in a very narrow repeating unit mass region of PEG. It is, therefore, a challenging task to correctly assign all of these peaks.

As demonstrated in the previous section, protonated ions can selectively be generated by depositing a sample on a preloaded layer of CHCA with appropriate co-matrixes. Compared with the selective formation of protonated ions, the selective formation of metal ion adducts for the PEG sample is much more straightforward. Even if the sample is pre-protonated, the protonated ions can completely be suppressed by sodium or potassium adducts via simply adding a certain amount of a suitable sodium or potassium salt and using the convenient dried-droplet sample deposition method.

The sorted MALDI mass spectra for the same PEG mixture with only protonated ions and sodium and potassium adducts are shown in Figure 3B, C, and D, respectively. Compared to Figure 3A, these spectra are greatly simplified and much easier to interpret because only one type of ions is present in each of these spectra. In Figure 3C and D, three series of PEG with different end groups can readily be identified. In contrast, only two series were observed in Figure 3B because H(OC_2_H_4_)_*n*_OH contains no amino end group and cannot be protonated by the proton transfer reactions of our method. Correlating the corresponding mass peaks in each spectrum allows unambiguous assignment of the ion peaks. For example, the mass peaks at *m*/*z* of 1409.9, 1431.9, and 1447.9 in Figure 3B, C, and D are respectively protonated ions and sodium and potassium adducts for molecules with the same formula of NH_2_C_3_H_6_–(OC_2_H_4_)_29_–OC_3_H_6_NH_2_. Interestingly, the mass of the end groups of M1 is 132 Da, which is equal to three PEG repeat units. Therefore, the mass peaks just discussed above could also theoretically be for cyclic PEG with the formula of (OC_2_H_4_)_32_. As cyclic PEG has no amino end groups and cannot be protonated with our method, the prominent peaks of [M1 + H]^+^ clearly indicate the presence of M1. Apparently, the sorted spectra will make the peak assignment not only much easier to perform but also more reliable as well.

In conclusion, for PEG samples, various ion peaks can be packed densely within a small repeating unit mass region of 44 Da (OC_2_H_4_), which makes the MALDI mass spectrum difficult to interpret. Disentangling these ions into three separate spectra with each spectrum containing only protonated ions and sodium and potassium adducts, respectively, can greatly simplify the interpretation of the MS results and allow unambiguous identification of the mass peaks. Selective formation of sodium or potassium adducts can simply be achieved by using the dried-droplet sample deposition method with NaTFA or KTFA as the co-matrix, while the selective formation of protonated ions can be achieved by carefully depositing the polymer sample on top of a preloaded layer of CHCA with ODA or a mixture of an ammonium salt and a tetrabutylammonium salt as the co-matrixes. In the latter case, the formation of sodium and potassium adducts can be inhibited by the co-matrix, while the protonated ions can be formed by proton transfer reactions from [ODA + H]^+^ or NH_4_^+^.

## References

[ref1] Tanaka K.; Waki H.; Ido Y.; Akita S.; Yoshida Y.; Yoshida T. Protein and Polymer Analyses up to m/z 100 000 by Laser Ionization Time-of-flight Mass Spectrometry. Rapid Commun. Mass Spectrum. 1988, 2 (8), 151–153. 10.1002/rcm.1290020802.

[ref2] Karas M.; Bachmann D.; Hillenkamp F. Influence of the Wavelength in High-Irradiance Ultraviolet. Anal. Chem. 1985, 57, 2935–2939. 10.1021/ac00291a042.

[ref3] Nielen M. W. F. MALDI Time-of-Flight Mass Spectrometry of Synthetic Polymers. Mass Spectrom. Reviews 1999, 18, 309–344. 10.1002/(SICI)1098-2787(1999)18:5<309::AID-MAS2>3.0.CO;2-L.

[ref4] Hillenkamp F.; Peter-Katalinić J. MALDI MS. A Practical Guide to Instrumentation, Method and Applications; Wiley-VCH: Weinheim, Germany, 2007.

[ref5] Cole R. B. Electrospray and MALDI Mass Spectrometry. Fundamentals, Instrumentation, Practicalities, and Biological Applications, 2nd ed.; Wiley: Hoboken, NJ, 2010.

[ref6] Yoo H.; Kim D.; Shin D.; Oh Y.; Lee S.; Lee J.; Choi Y.; Lee S.; Lee K.; Kimb Y.; Cho K. Recent Developments in Pre-treatment and Analytical Techniques for Synthetic Polymers by MALDI-TOF Mass Spectrometry. Anal. Methods 2020, 12, 5767–5800. 10.1039/D0AY01729A. 33241791

[ref7] Hiemenz P. C. Polymer Chemistry; Marcel Dekker: New York, 1984.

[ref8] Zenobi R.; Knochenmuss R. Ion Formation in MALDI Mass Spectrometry. Mass Spectrom. Reviews 1998, 17, 337–366. 10.1002/(SICI)1098-2787(1998)17:5<337::AID-MAS2>3.0.CO;2-S.

[ref9] Wu K. J.; Odom R. W. Characterizing Synthetic Polymers by MALDI MS. Anal. Chem. 1998, 70, 456A–461A. 10.1021/ac981910q. 9666717

[ref10] Urban P. L.; Amantonico A.; Zenobi R. Lab-on-a-Plate: Extending the Functionality of MALDI-MS and LDI-MS Targets. Mass Spectrom. Reviews 2011, 30, 435–478. 10.1002/mas.20288. 21254192

[ref11] Murray K. K. DNA Sequencing by Mass Spectrometry. J. Mass Spectrom. 1996, 31, 1203–1215. 10.1002/(SICI)1096-9888(199611)31:11<1203::AID-JMS445>3.0.CO;2-3. 8946729

[ref12] Li Y. C. L.; Cheng S.-w.; Chan T.-W. D. Evaluation of Ammonium Salts as Co-matrices for Matrix-assisted Laser Desorption/Ionization Mass Spectrometry of Oligonucleotides. Rapid Commun. Mass Spectrom. 1998, 12, 993–998. 10.1002/(SICI)1097-0231(19980815)12:15<993::AID-RCM273>3.0.CO;2-4.

[ref13] Distler A. M.; Allison J. Improved MALDI-MS Analysis of Oligonucleotides through the Use of Fucose as a Matrix Additive. Anal. Chem. 2001, 73, 5000–5003. 10.1021/ac015550+. 11681479

[ref14] Knochenmuss R.; Zenobi R. MALDI Ionization: The Role of In-Plume Processes. Chem. Rev. 2003, 103, 441–452. 10.1021/cr0103773. 12580638

[ref15] Lou X.; van Dongen J. L.J.; Vekemans J. A. J. M.; Meijer E. W. Matrix suppression and analyte suppression effects of quaternary ammonium salts in matrix-assisted laser desorption/ionization time-of-flight mass spectrometry. An investigation of suppression mechanism. Rapid Commun. Mass Spectrom. 2009, 23, 3077 10.1002/rcm.4224. 19705379

[ref16] Lou X.; van Dongen J. L. J.; Milroy L.-G.; Meijer E. W. Generation of gas-phase ions from charged clusters: an important ionization step causing suppression of matrix and analyte ions in matrix-assisted laser desorption/ionization mass spectrometry. Rapid Commun. Mass Spectrom. 2016, 30, 2628–2634. 10.1002/rcm.7741. 27643391

[ref17] Gusev A. I.; Wilkinson W. R.; Proctor A.; Hercules D. M. Improvement of Signal Reproducibility and Matrix/Comatrix Effects in MALDI Analysis. Anal. Chem. 1995, 67, 1034–1041. 10.1021/ac00102a003.

[ref18] Asara J. M.; Allison J. Enhanced Detection of Phosphopeptides in Matrix-Assisted Laser Desorption/Ionization Mass Spectrometry Using Ammonium Salts. J. Am. Soc. Mass Spectrom. 1999, 10, 35–44. 10.1016/S1044-0305(98)00129-9. 9888183

[ref19] Lou X.; de Waal B. F. M.; Lech-Gustav Milroy L.-G.; van Dongen J. L. J. A sample preparation method for recovering suppressed analyte ions in MALDI TOF MS. J. Mass Spectrom. 2015, 50, 766–770. 10.1002/jms.3587. 26259660

[ref20] Guo Z.; Zhang Q.; Zou H.; Guo B.; Ni J. A method for the analysis of low-mass molecules by MALDI-TOF mass spectrometry. Anal. Chem. 2002, 74, 1637 10.1021/ac010979m. 12033256

